# Association of Environment With the Risk of Developing Psychotic Disorders in Rural Populations: Findings from the Social Epidemiology of Psychoses in East Anglia Study

**DOI:** 10.1001/jamapsychiatry.2017.3582

**Published:** 2017-11-29

**Authors:** Lucy Richardson, Yasir Hameed, Jesus Perez, Peter B. Jones, James B. Kirkbride

**Affiliations:** 1 PsyLife Group, Division of Psychiatry, University College London, London, England; 2 Norfolk and Suffolk National Health Service Foundation Trust, Hellesdon Hospital, Norwich, Norfolk, England; 3 Department of Psychiatry, University of Cambridge, Cambridge, England; 4 Cambridgeshire and Peterborough National Health Service Foundation Trust, Cambridge, England; 4 Department of Neuroscience, Instituto de Investigación Biomédica de Salamanca (IBSAL), University of Salamanca, Spain

## Abstract

**Importance:**

Social determinants are important risk factors for the development of first-episode psychosis (FEP); their effects in rural areas are largely unknown.

**Objective:**

To investigate neighborhood-level factors associated with FEP in a large, predominantly rural population-based cohort.

**Design, Setting, and Participants:**

This study extracted data on referrals for treatment of potential FEP at 6 Early-Intervention Psychosis services from the Social Epidemiology of Psychoses in East Anglia naturalistic cohort study data set, which covered a population of more than 2 million people in a rural area in the East of England for a period of 3.5 years. All individuals aged 16 to 35 years who presented to Early-Intervention Psychosis services and met diagnostic criteria for first episodes of nonaffective psychoses and affective psychoses (*International Statistical Classification of Diseases and Related Health Problems, Tenth Revision* diagnostic codes F20-33) were included (n = 631). Persons whose disorders had an organic basis (diagnostic codes F06.X) and those meeting the criteria for substance-induced psychosis (diagnostic codes F1X.5) were excluded. We derived 4 neighborhood-level exposures from a routine population data set using exploratory factor analysis (racial/ethnic diversity, deprivation, urbanicity, and social isolation) and investigated intragroup racial/ethnic density and fragmentation.

**Main Outcomes and Measures:**

Multilevel Poisson regression was performed to determine associations between incidence rates and neighborhood-level factors, after adjustment for individual factors. Results were reported as incidence rate ratios (IRRs).

**Results:**

The study included 631 participants who met criteria for FEP and whose median age at first contact was 23.8 years (interquartile range, 19.6-27.6 years); 416 of 631 (65.9%) were male. Crude incidence of FEP was calculated as 31.2 per 100 000 person-years (95% CI, 28.9-33.7). Incidence varied significantly between neighborhoods after adjustment for age, sex, race/ethnicity, and socioeconomic status. For nonaffective psychoses, incidence was higher in neighborhoods that were more economically deprived (IRR, 1.13; 95% CI, 1.06-1.20) and socially isolated (IRR, 1.11; 95% CI, 1.04-1.19). It was lower in more racially/ethnically diverse neighborhoods (IRR, 0.94; 95% CI, 0.87-1.00). Higher intragroup racial/ethnic density (IRR, 0.97; 95% CI, 0.94-1.00) and lower intragroup racial/ethnic fragmentation (IRR, 0.98; 95% CI, 0.96-1.00) were associated with a reduced risk of affective psychosis.

**Conclusions and Relevance:**

Spatial variation in the incidence of nonaffective and affective psychotic disorders exists in rural areas. This suggests that the social environment contributes to psychosis risk across the rural-urban gradient.

## Key Points

**Question:**

Is the social and built environment associated with the risk of developing psychotic disorders in rural populations?

**Findings:**

In this cohort study of 631 persons with first-episode psychosis, significant variation was found in the incidence of nonaffective and affective psychotic disorders between rural neighborhoods. Nonaffective psychoses occurred more frequently in more economically deprived, more socially isolated, and less racially/ethnically diverse communities, while greater intragroup racial/ethnic density and less racial/ethnic fragmentation were associated with lower rates of affective psychoses after taking into account individual factors.

**Meaning:**

Exposure to social adversities potentially influences psychosis risk across the rural-urban continuum.

## Teaser

This cohort study investigated neighborhood-level factors associated with first-episode psychosis in a predominantly rural population in the East of England.

## Introduction

People born and raised in urban environments face elevated risk of psychotic disorders such as schizophrenia compared with people living in rural areas.^1^ This is not explained by family history of psychiatric illness or other potential confounders, such as paternal age,^2^ age,^3^ sex,^3^ or race/ethnicity.^5^ Several socioenvironmental factors have been proposed to explain the variation, including deprivation (a single measure that combines income, employment, disability, education, housing, environment, and crime into an approximation of the overall socioeconomic prosperity of an area),^6^ inequality (a quantification of relative, rather than absolute, deprivation),^9^ social fragmentation (a lack of social connections between individuals of a given geographic area),^6^ and racial/ethnic density (a measure of the degree to which people of the same racial/ethnic origin live together in a given geographic location).^9^ However, much less research has investigated whether risk in rural areas varies according to exposure to such social adversities, in part because of the difficulty of conducting studies of rare outcomes in sparse population settings. A collection of studies from rural Ireland^16^ has suggested that rates may vary by deprivation. This finding was replicated in our 2017 study in rural England.^4^ However, to our knowledge, no study to date has investigated whether a broader array of socioenvironmental risk factors or aspects of the physical and built environment are associated with psychosis risk in rural populations. Furthermore, while nonaffective psychoses tend to show more variation at the neighborhood level than affective psychoses,^3^ this issue has yet to be quantified in rural settings to our knowledge.

We used epidemiological data from a large, naturalistic cohort of participants who presented to Early Intervention in Psychosis (EIP) services with first-episode psychosis (FEP) to investigate whether psychosis incidence varied according to neighborhood-level social and environmental factors in a predominantly rural setting in the East of England. We hypothesized that, after adjusting for individual-level factors, (1) nonaffective psychoses would show spatial variation in incidence across different neighborhoods; (2) affective psychoses would show less spatial variation; (3) incidence rates would be higher in more deprived, more socially isolated, and more urban parts of the study region; (4) neighborhood-level racial/ethnic density would be inversely associated with the relative risk of FEP in black and minority racial/ethnic groups; and (5) racial/ethnic fragmentation would be inversely associated with the relative risk of FEP in black and minority racial/ethnic groups.

## Methods

### Setting

We used data from the Social Epidemiology of Psychoses in East Anglia study,^4^ which ascertained all incidence cases presenting to EIP services during 3.5 years. The catchment area contained 2.4 million people in 2011 and was predominantly rural (median population per square mile, 587.8; interquartile range, 208.9-4653.4) compared with the rest of England (median population per square mile, 3645.6; interquartile range, 573.1-8976.3) (Mann-Whitney *U*, 12.1; *P* < .001).

### Ethics

The Cambridgeshire III Local Research Ethics Committee (09/H0309/39) granted ethical approval for this study to collect anonymized statistical data without informed consent. This was consistent with the use of statistical data in the public interest as specified in the United Kingdom Data Protection Act of 1998.^27^

### Case Ascertainment

In England, EIP services are the sole referral point for all people with suspected FEP. Services in the catchment area worked closely with primary, secondary, and tertiary health care services, including general practitioners, other mental health facilities in the National Health Service, schools, and universities; we have previously shown case ascertainment via these services leads to unbiased estimates of incidence.^4^ We followed up with all participants accepted by EIP services until they had received 3 years of care or were discharged from services (if the discharge occurred before 3 years had elapsed). In this study, we included participants if, at first referral, they were aged 16 to 35 years; resided in the catchment area (excluding those of no fixed abode); had no previous contact with health services for psychosis and no previous treatment with antipsychotic medication for more than 6 months; and were presenting to EIP services with clinical evidence of a FEP, per the criteria of diagosis found in *International Statistical Classification of Diseases and Related Health Problems, Tenth Revision (ICD-10)* (codes F20-33). Patients with comorbid moderate or severe learning disabilities, an organic basis to disorder, or substance-induced psychosis (*ICD-10* code F1X.5) were excluded from the study.

Participants who met the inclusion criteria were subsequently assessed to confirm a diagnosis of *ICD-10* psychotic disorder via standardized case note review according to the Operational Criteria Checklist for Psychotic Illness,^21^ a reliable diagnostic instrument^22^ with good interrater reliability.^4^ We classified participants according to 3 outcomes, as described in detail elsewhere^4^: first, by all nonorganic psychotic disorders (*ICD-10* codes F20-33), then into groups of nonaffective psychotic disorders (F20-29) and affective psychotic disorders (F30-33).

### Sociodemographic Variables

At first referral, we used a standardized form to collect basic sociodemographic data on all participants, including age at referral, sex, race/ethnicity, socioeconomic status (SES), and neighborhood of residence. Age was treated as categorical (16-19, 20-24, 25-29, and 30-35 years). Participants self-reported 1 of 18 racial/ethnic categories from the 2011 census,^23^ which we collapsed into 11 analytical groups (white British, white other, mixed white and black Caribbean, mixed other, Indian, Pakistani, Bangladeshi, black African, black Caribbean, Arab, and any other racial/ethnic group). Of these, all groups except the white British were also grouped into a black and minority racial/ethnic group for the purposes of descriptive data reporting. Socioeconomic status was based on participant occupation at presentation (per Office for National Statistics decision rules^24^) and was categorized into 5 groups: people in professional and managerial occupations; those in intermediate occupations, small employers, and self-employed persons; lower supervisory and technical employees; those in semiroutine and routine jobs; and students, unemployed persons, and those who had never worked.

### Neighborhood-Level Exposures

The study region was organized into 530 administrative neighborhoods known as statistical wards, which formed the area-level unit of analysis in our study (median population, 3992 people; interquartile range, 2426-5935). We included a comprehensive set of 29 social and built environment variables—putatively relevant to the causal mechanisms of psychosis—from routine data sources (eAppendix and eTable 1 in the Supplement). All variables were entered into an exploratory factor analysis with Varimax rotation to identify distinct neighborhood-level socioenvironmental exposures. We omitted 7 neighborhood variables with poor psychometric properties from the final factor analysis (eTable 1 and eFigure in the Supplement). Factor retention was identified by examination of a scree plot, with substantive item loadings (≥0.4) taken to indicate important associations. We extracted neighborhood-level factor scores for each factor and standardized to have a mean of 0 and SD of 1. We created categorical versions of each factor to test for possible nonlinear associations with our outcomes, which involved classifying neighborhoods as low (≤25th percentile), medium (26th-75th percentile) and high (≥76th percentile) on each exposure.

Separately, we estimated neighborhood-level intragroup racial/ethnic density and fragmentation, because we were interested in their independent effects on psychosis risk. Intragroup racial/ethnic density was defined as the proportion of the racial/ethnic group of a given participant in each neighborhood.^9^ Intragroup racial/ethnic fragmentation was defined as the distribution of the racial/ethnic group of a given participant across a neighborhood; this was measured using Peach Index of Dissimilarity,^26^ which is based on the percentage of people from a given racial/ethnic group who would have to move to another part of the same neighborhood to live in a totally integrated spatial pattern with the remainder of the total population. Higher percentages indicated higher segregation, and therefore lower intragroup racial/ethnic fragmentation.

### Population at Risk

The population at risk was estimated from the 2011 census of Great Britain,^23^ which was collected close to the midpoint of case ascertainment for the present study. Data were stratified by age group, sex, race/ethnicity, and SES. The total was multiplied by 3.5 years to estimate person-years at risk.

### Statistical Analysis

First, we reported descriptive statistics of the sample according to neighborhood-level exposures, with 2-tailed χ^2^ tests to analyze differences between each psychosis outcome and the population at risk. Second, we characterized variations in the social and built environment using factors derived from the exploratory factor analysis. Third, using these derived neighborhood-level exposures, we conducted multilevel Poisson regression with random intercepts to investigate the effects of factors on incidence for each outcome, after controlling for individual-level age, sex, race/ethnicity, and SES. Modeling proceeded as follows: null (empty) models were created to quantify initial variance in incidence at the neighborhood level. Individual-level a priori confounders were then added to the model to quantify changes to this variance. Finally, we constructed multivariable models by adding neighborhood-level exposures as fixed effects in the order of their strength of association with a given outcome, as reported in univariable analyses. These analyses were based on Akaike Information Criterion, where lower scores indicated better model fit. Model building was tested via the likelihood ratio test (LRT). In our final models, we tested for departure from linearity of our continuous neighborhood-level exposures by substituting the equivalent categorical constructs (as described above) and comparing Akaike Information Criterion scores between these nonnested models.

We tested for statistical interaction between individual-level race/ethnicity and neighborhood-level intragroup racial/ethnic density or fragmentation to investigate whether their effects on psychosis risk differed by racial/ethnic group. We checked final models against Poisson regression assumptions (overdispersion, zero inflation) and found that they were not violated (eTable 2 in the Supplement). The present study reports incidence rate ratios (IRRs) that compare relative difference in psychosis incidence between groups separated by 1 or more SD with respect to the socioenvironmental factors under exploration. In addition, 95% confidence intervals (CI) are reported. All statistical tests were set at a significance level of *P* < .05.

## Results

### Sample Demographics and Crude Incidence Rates

Of the 631 individuals who met inclusion criteria for psychotic disorders under *ICD-10* codes F20 through 33 (Table 1), 573 (87.2%) received diagnoses of nonaffective psychoses (*ICD-10* codes F20-29) and 84 (12.8%) received diagnoses of affective psychoses (*ICD-10* codes F30-33). This corresponded to crude incidence rates of 31.2 new cases per 100 000 person-years for all psychotic disorders (95% CI, 28.9-33.7), 27.1 for nonaffective psychoses (95% CI, 24.9-29.5), and 4.1 for affective psychoses (95% CI, 3.3-5.1). Median age at first contact was 23.8 years (interquartile range, 19.6-27.6 years). Compared with the population at risk, participants with FEP were more likely to be male, younger, unemployed, of lower SES, and from a black and minority racial/ethnic group (*P* < .001; all comparisons are detailed in Table 1). Participants from these backgrounds had lower intragroup racial/ethnic density, were more racially/ethnically fragmented, and lived in more racially/ethnically diverse neighborhoods than the white British population (eTable 3 in the Supplement).

**Table 1.** Sociodemographic Characteristics of Study Participants

Variable	No. (%)				*P *Value (χ^2^)^**d**^
	All FEP^a^	Nonaffective Psychoses^b^	Affective Psychoses^c^	Denominator (Person-years at Risk)	
Total	631 (100.0)	548 (100.0)	83 (100.0)	2 021 794 (100.0)	
Sex					< .001 (χ^2^_1_=55.8)
Male	416 (65.9)	369 (67.3)	47 (56.6)	1 032 306 (51.1)	
Female	215 (34.1)	179 (32.7)	36 (43.4)	989 488 (48.9)	
Age group, y					< .001 (χ^2^_2_=98.9)
16-24	398 (63.1)	347 (63.3)	51 (61.5)	896 405 (44.3)	
25-29	137 (21.7)	114 (20.8)	23 (27.7)	525 134 (26.0)	
30-35	96 (15.2)	87 (15.9)	9 (10.8)	600 255 (29.7)	
Race/ethnicity^e^					< .001 (χ^2^_1_=12.7)
White British	471 (74.6)	418 (76.3)	53 (63.9)	1 623 285 (80.3)	
White other	62 (9.8)	50 (9.1)	12 (14.5)	207 165 (10.2)	
Mixed white and black Caribbean	7 (1.1)	5 (0.9)	2 (2.4)	13 100 (0.6)	
Mixed other	17 (2.7)	11 (2.0)	6 (7.2)	30 927 (1.5)	
Indian	2 (0.3)	2 (0.4)	NA	27 911 (1.4)	
Pakistani	16 (2.5)	13 (2.4)	3 (3.6)	20 126 (1.0)	
Bangladeshi	6 (1.0)	5 (0.9)	1 (1.2)	8403 (0.4)	
Black African	22 (3.5)	21 (3.8)	1 (1.2)	17 193 (0.9)	
Black Caribbean	9 (1.4)	6 (1.1)	3 (3.6)	5973 (0.3)	
Arab	4 (0.6)	4 (0.7)	NA	4838 (0.2)	
Any other racial/ethnic group	15 (2.4)	13 (2.4)	2 (2.4)	62 875 (3.1)	
Socioeconomic status					< .001 (χ^2^_5_=144.5)
Professional and managerial	69 (10.9)	57 (10.4)	12 (14.5)	493 719 (24.4)	
Intermediate	48 (7.6)	39 (7.1)	9 (10.8)	229 304 (11.3)	
Small employers and self-employed	28 (4.4)	24 (4.4)	4 (4.8)	104 509 (5.2)	
Lower supervisory and technical	15 (2.4)	14 (2.6)	1 (1.2)	154 560 (7.6)	
Semiroutine and routine	235 (37.2)	206 (37.6)	29 (34.9)	514 275 (25.4)	
Unemployed, student, and those who have never worked	236 (37.4)	208 (38.0)	28 (33.7)	525 429 (26.0)	

Abbreviations: FEP, first-episode psychosis; *ICD-10*, *International Statistical Classification of Diseases and Related Health Problems, Tenth Revision*; NA, not applicable.

^a^ First-episode psychosis includes all diagnoses under *ICD-10* codes F20 through F33.

^b^ Nonaffective psychoses includes all diagnoses under *ICD-10* codes F30 through F33.

^c^ Affective psychoses includes all diagnoses under *ICD-10* codes F20 through F29.

^d^ χ^2^ Test reports the difference in the distribution of all FEP cases and the denominator population for each variable; subscript numeral denotes degrees of freedom.

^e^ It was not possible to conduct χ^2^ or Fisher exact test on this variable owing to sparse data in some cells, so a binary variable was created to test differences between the white British vs black and minority racial/ethnic groups using a χ^2^ test.

### Exploratory Factor Analysis

Our exploratory factor analysis (eTable 4 in the Supplement) suggested that 4 latent constructs of socioenvironmental adversity provided the optimum factor solution, informed by inspection of a scree plot (eFigure in the Supplement). These 4 factors explained 90% of total variance in 22 neighborhood items. We termed the first factor *racial/ethnic diversity* because it included several items related to the racial/ethnic composition of a neighborhood, including the proportion of people whose identity was ascribed to a national identity from a place other than the United Kingdom, recent overseas immigration, an item-level estimate of racial/ethnic diversity, and population turnover (inmigration to and outmigration from the neighborhood). We termed the second factor *deprivation* because several items related to socioeconomic conditions loaded strongly onto this dimension, including a negative association with inequality. Our third factor was a construct of *urbanicity*, with a strong positive loading on population density, and strong negative loadings on green space, nondomestic buildings, and travel times. Our final factor, *social isolation*, incorporated items predominantly indicating social connectedness within a neighborhood, including single-person households, noncohabiting people, population turnover, and lack of car ownership. The spatial distribution of these neighborhood-level constructs varied across the region (Figure; for data sources, see eTable 1 in the Supplement, and for details of items loading onto each construct, see eTable 4 in the Supplement).

**Figure.**
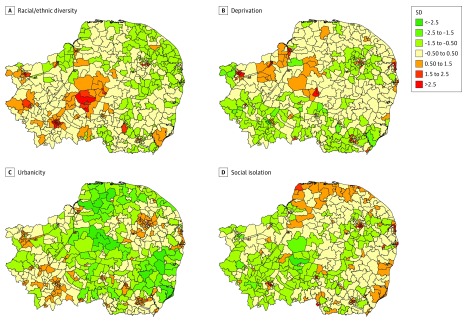
Variation in Socioenvironmental Exposures Identified From Exploratory Factor Analysis Across 530 Neighborhoods in the Social Epidemiology of Psychoses in East Anglia (SEPEA) East of England Catchment Area The 4 panels show variation across 530 neighborhoods in the SEPEA catchment area in constructs of racial/ethnic diversity (pronounced in the center and west of the study region across low and high areas of population density) (A); deprivation (predominant in the north and east of the region) (B); urbanicity (closely matched to population density) (C); and social isolation (found in the north and east of the region and in Cambridge city [southwest]) (D). Colors denote the number of standard deviations above or below the mean for the whole catchment area. For all constructs, scores across the study region were standardized to have a mean of 0 and SD of 1.

### Multilevel Modeling

#### Neighborhood Variance for All Outcomes

A null multilevel model indicated statistically significant neighborhood-level variance in all FEP outcomes, which showed little attenuation after control for individual-level age, sex, race/ethnicity, and SES (Table 2). Inclusion of neighborhood-level exposures in our final models (described below) reduced this variance to nonsignificance for nonaffective psychoses (SD, 0.04; 95% CI, 0.01-0.33; *P* = .15) and affective psychoses (SD, 0.58; 95% CI, 0.19-1.78; *P* = .09) separately, although residual variance remained when all FEP psychoses were considered as a single outcome (SD, 0.07; 95% CI, 0.02-0.22; *P* = .02).

**Table 2.** Variance in First-Episode Psychosis as Accounted for by Neighborhood Characteristics

Outcome^**a**^	Model					
	Null		Individual Characteristics^**b**^		Neighborhood Characteristics^**c**^	
	Random Effect (95% CI)	*P* Value	Random Effect (95% CI)	*P* Value	Random Effect (95% CI)	*P* Value
All psychoses	0.12 (0.05-0.25)	.001	0.12 (0.06-0.25)	.001	0.07 (0.02-0.22)	.02
Nonaffective	0.11 (0.04-0.27)	.01	0.11 (0.05-0.26)	.003	0.04 (0.01-0.33)	.15
Affective	0.63 (0.23-1.72)	.01	0.60 (0.21-1.75)	.01	0.58 (0.19-1.78)	.09

Abbreviation: *ICD-10, International Statistical Classification of Diseases and Related Health Problems, Tenth Revision*.

^a^ All psychoses includes all diagnoses under *ICD-10* codes F20 through F33. Affective psychosis includes all diagnoses under *ICD-10* codes F20 through F29, and nonaffective psychosis included all diagnoses under *ICD-10* codes F30 through F33.

^b^ Individual factors: age, socioeconomic status, sex. and race/ethnicity.

^c^ Adjusted for individual-level age, socioeconomic status, sex, and race/ethnicity. Neighborhood-level factors were statistically significant factors included in final model: racial/ethnic density, racial/ethnic diversity, deprivation, urbanicity, and social isolation.

#### All First-Episode Psychosis

In unadjusted Poisson regression, we observed associations between FEP and racial/ethnic diversity, deprivation, and social isolation (Table 3). After adjustment for individual-level factors in our final multilevel model, 3 neighborhood-level exposures were associated with psychosis risk: deprivation (with a change in 1 SD from the mean: IRR, 1.12; 95% CI, 1.06-1.19), social isolation (IRR, 1.09; 95% CI, 1.03-1.16), and urbanicity (IRR, 1.11; 95% CI, 1.00-1.23) (*P* = .04). There was no evidence of interaction between race/ethnicity and racial/ethnic density (LRT χ^2^_10_ = 10.8; *P* = .37) or racial/ethnic fragmentation(LRT χ^2^_10_ = 14.0; *P* = .17).

**Table 3.** Association Between Neighborhood-Level Exposures and Incidence Rate Ratios of Psychotic Disorders

Neighborhood variable^**a**^^,^^**b**^^,^^**c**^	Univariable Analysis		Multivariable Analysis^**d**^	
	IRR (95% CI)	AIC	IRR (95% CI)	LRT *P* Value
All psychoses				
Racial/ethnic fragmentation	1.01 (1.01-1.02)	6081.0	1.00 (0.99-1.01)	.40
Racial/ethnic density	0.996 (0.99-1.00)	6078.8	1.00 (0.99-1.01)	.86
Racial/ethnic diversity	1.00 (0.94-1.06)	6092.0	0.95 (0.89-1.02)	.14
Deprivation	1.14 (1.08-1.21)	6073.5	1.12 (1.06-1.19)	<.001
Urbanicity	1.09 (0.98-1.21)	6089.5	1.11 (1.00-1.23)	.04
Social isolation	1.09 (1.02-1.16)	6085.0	1.09 (1.03-1.16)	.006
Nonaffective psychoses				
Racial/ethnic fragmentation	1.01 (1.01-1.02)	5401.1	1.00 (0.99-1.01)	.42
Racial/ethnic density	0.996 (0.99-1.00)	5405.9	0.99 (0.98-1.00)	.23
Racial/ethnic diversity	0.98 (0.92-1.05)	5411.6	0.94 (0.87-1.00)	.05
Deprivation	1.15 (1.09-1.23)	5392.6	1.13 (1.06-1.20)	<.001
Urbanicity	1.08 (0.97-1.20)	5410.1	1.09 (0.98-1.21)	.10
Social isolation	1.09 (1.02-1.17)	5405.1	1.11 (1.04-1.19)	.002
Affective psychoses				
Racial/ethnic fragmentation	1.01 (0.98-1.03)	1165.7	0.97 (0.94-1.00)	.03
Racial/ethnic density	0.989 (0.98-1.00)	1152.3	0.98 (0.96-1.00)	.05
Racial/ethnic diversity	1.10 (0.94-1.30)	1164.7	0.87 (0.71-1.06)	.17
Deprivation	1.07 (0.90-1.28)	1165.4	1.01 (0.85-1.20)	.93
Urbanicity	1.12 (0.84-1.50)	1165.4	1.03 (0.76-1.40)	.84
Social isolation	1.08 (0.91-1.28)	1165.3	1.04 (0.88-1.24)	.65

Abbreviations: AIC, Akaike Information Criterion; *ICD-10*, *International Statistical Classification of Diseases and Related Health Problems, Tenth Revision*; IRR, incident rate ratio; LRT, likelihood ratio test; z: *z* standardized, where IRR corresponds to change in incidence associated with a 1 SD change in exposure.

^a^ Diagnostic categories include all psychoses (all diagnoses under *ICD-10* codes F20-F33); affective psychoses (*ICD-10* codes F20-F29), and nonaffective psychoses (*ICD-10* codes F30-F33).

^b^ Variables racial/ethnic diversity, deprivation, urbanicity, and social isolation were derived from exploratory factor analysis.

^c^ All neighborhood variables are presented via *z* (standard) scores, except racial/ethnic fragmentation and race/ethnicity, which are presented as percentages.

^d^ The final model adjusted for individual-level age, socioeconomic status, sex, race/ethnicity, and all statistically significant neighborhood variables shown in the multivariable analysis columns for each outcome.

#### Nonaffective Psychoses

We observed similar results for nonaffective psychoses as a separate outcome (Table 3), with greater deprivation (IRR, 1.13; 95% CI, 1.06-1.20) and social isolation (IRR, 1.11; 95% CI, 1.04-1.19) associated with higher incidence in our final model. There was also some evidence that greater racial/ethnic diversity was associated with lower incidence (IRR, 0.94; 95% CI, 0.87-1.00; LRT *P* = .05); urbanicity did not improve the final model (LRT χ^2^_1_ = 2.7; *P* = .10). There was no interaction between race/ethnicity and racial/ethnic density (LRT χ^2^_10_ = 11.0; *P* = .36) or racial/ethnic fragmentation (LRT χ^2^_10_ = 6.9; *P* = .73).

#### Affective Psychoses

In our final model for affective psychoses, lower incidence rates were independently associated with greater intragroup racial/ethnic density (IRR, 0.98; 95% CI, 0.96-1.00) and lower intragroup racial/ethnic fragmentation (IRR, 0.97; 95% CI, 0.94-1.00) (Table 3). Together, these 2 factors could be described as higher segregation of racial/ethnic groups. There was no interaction between race/ethnicity and racial/ethnic density (LRT χ^2^_10_ = 4.4; *P* = .93), or race/ethnicity and race/ethnic fragmentation (LRT χ^2^_10_ = 17.4; *P* = .07). Nevertheless, further inspection suggested that while lower fragmentation was associated with reduced incidence of affective psychoses for most groups (IRR, 0.95; 95% CI, 0.91-0.98), this pattern was reversed for people of mixed (other) racial/ethnic backgrounds (IRR, 1.09; 95% CI, 1.02-1.16).

#### Categorical Neighborhood Variables

For all outcomes, there was no evidence that the observed pattern of associations with neighborhood-level exposures showed a departure from linearity when our final models were refitted with categorical variables.

## Discussion

### Interpretation of Principal Findings

To our knowledge, this is the largest epidemiological study of psychotic disorder risk in rural environments, and we have provided strong evidence of neighborhood-level variation in nonaffective and affective psychotic disorders that cannot be explained by individual-level factors. This variance was associated with signs of the social and built environment, including socioeconomic deprivation, urbanicity, racial/ethnic density, and racial/ethnic fragmentation, where effects on risk were generally similar for all racial/ethnic groups. Unlike previous studies in more urban populations and in contrast to our hypothesis, we found significant variation in affective psychotic disorders; this was largely explained by neighborhood-level racial/ethnic composition. Our results suggest that social and physical attributes of neighborhoods influence the spatial patterning of psychotic disorders across the rural-urban continuum.

Our research extends well-established findings that urban living is associated with psychosis risk. Like previous studies,^1^ we found higher FEP rates in more urban parts of our study region. However, our study setting was predominantly rural, and here, rates also varied by deprivation, social isolation, and neighborhood racial/ethnic composition. Deprivation has previously been linked to FEP risk in rural Ireland,^18^ although, unlike the present investigation, that study did not control for individual-level SES. Importantly, our findings suggest that the social environment may affect the incidence of psychotic disorders across the rural-urban gradient. This is important because it implies that some of the social determinants of psychosis incidence—including exposure to deprivation or social isolation—are risk factors regardless of where they occur; nonetheless, such exposures are likely to have greater effect in urban areas by virtue of their population size and structure, given that young people and people from racial/ethnic minority backgrounds—both of whom are at greater psychosis risk of psychotic disorders^4^—are more likely to live in cities.

The mechanisms through which the social environment acts on psychosis risk are worthy of further consideration. People living in more deprived areas might also be exposed to other adverse living conditions, such as high crime rates, exposure to violence, social stress, and lower quality or quantity of local services, including housing and other amenities.^15^ Similarly, neighborhoods with higher social isolation may fail to buffer people from the negative consequences of stress after exposure to social adversity, potentially leaving them vulnerable to developing psychosis^28^; corollary evidence of such a buffering effect exists after exposure to childhood trauma.^30^ Social isolation and fragmentation have also been associated with psychotic disorders in nationwide studies^10^ and more urban populations.^32^

If social isolation is a risk factor for psychotic disorders, then factors that buffer its effect—including neighborhood racial/ethnic composition—might be protective. Here, greater racial/ethnic diversity was associated with reduced incidence of nonaffective psychoses; this might be an indication of bridging social capital (ie, connections, support, and trust between different groups) and suggests the benefit of promoting better racial/ethnic integration and social support in rural communities. These results accord with a previous finding on FEP risk and racial/ethnic integration among black Caribbean communities in East London.^9^ Nonetheless, most previous studies have shown that higher intragroup racial/ethnic density, a possible sign of bonding social capital (ie, connections, support and trust within a particular group), also reduces psychosis risk,^9^ but we only observed this association in people with affective psychoses. Our results with respect to affective psychoses were novel, given that other studies have predominantly failed to observe variance in incidence at the neighborhood level.^1^ In rural England—where black and minority racial/ethnic groups represent a small proportion of the total population (eTable 3 in the Supplement)—bridging social capital may become more relevant to mitigating social stress than bonding social capital, given more limited opportunities to interact with people from one’s own racial/ethnic minority group in the immediate environment.

### Methodological Considerations

Our study had several strengths. We used epidemiological data from a large, naturalistic cohort study using operationalized criteria to identify cases. We excluded people with an organic or substance-induced basis to their disorder and used broad psychotic phenotypes as our outcomes to minimize diagnostic bias. The sample was sufficiently large and diverse to consider the role of several environmental factors, including racial/ethnic diversity and, to our knowledge for the first time in relation to psychosis, aspects of the built environment. Here, our construct of urbanicity, which was positively associated with FEP incidence, included population density and novel indicators of the built environment, including green space, non-residential buildings, and travel time statistics. This gave a more precise estimation of urbanicity than previously used.

### Limitations

We note some important limitations of our study. Case ascertainment was based on presentation to EIP services in the East of England. While these services were engaged actively in outreach and were the sole referral point for young people with suspected psychotic symptoms, we cannot exclude the possibility that some incident cases were missed, given that we were unable to conduct a leakage study. It is unclear whether this would have differentiated places; for example, the current evidence is equivocal as to whether duration of untreated psychosis—a possible indication of delayed ascertainment—is associated with distance to services.^36^ Nevertheless, our previous research^4^ demonstrates that the magnitude and patterns of incidence overall, and by age, sex, race/ethnicity, and SES are in line with rates from other studies.^38^ The low incidence of affective psychoses, particularly compared with nonaffective psychoses, may have affected statistical power to detect some associations, although we observed neighborhood-level variation on this outcome. Similarly, measures of intragroup neighborhood racial/ethnic density and fragmentation were based on small numbers, which may have limited power to detect cross-level interactions with race/ethnicity. We were unable to adjust for some important potential confounders, including family history of psychosis,^39^ marital status,^11^ and prior substance use, because these data were not available for the denominator (census) population. We could only control for 1 aspect of SES, which was occupation; this could have led to some residual confounding at the individual level. Exposure and outcome data were collected contemporaneously, and, as such, we cannot exclude the potential for reverse causality. Further, given the risk of ecological fallacy, we cannot definitively infer that individuals living in neighborhoods with high levels of the exposures included in our study were directly exposed to them. In general, while neighborhood associations were robust, they were small in magnitude, typically resulting in about a 10% change in incidence associated with a change in exposure 1 SD in size.

## Conclusions

We found evidence of variation in the incidence of FEP across the rural-urban continuum, associated with deprivation, social isolation, and racial/ethnic composition. Social adversities, or failure to assuage the negative consequences of such adversities,^40^ may increase risk,^39^ but carefully designed longitudinal studies are required to determine causality.

## Notes

Supplement. **eAppendix.** Online-only text. **eFigure.** Scree plot following exploratory factor analysis. **eTable 1.** Variables included in the exploratory factor analysis. **eTable 2.** Over-dispersion data for each outcome. **eTable 3.** Neighborhood-level descriptive characteristics of racial/ethnic density, fragmentation and diversity, by racial/ethnic group. **eTable 4.** Rotated factor loadings following EFA.
