# Supplementary material for: Association of Environment With the Risk of Developing Psychotic Disorders in Rural Populations: Findings from the Social Epidemiology of Psychoses in East Anglia Study
Source: JAMA Psychiatry. 2017 Nov 29;75(1):75–83. doi: 10.1001/jamapsychiatry.2017.3582 (PMC5833554; doi:10.1001/jamapsychiatry.2017.3582)
Supplement: Supplement. — eAppendix. Online-only text. eFigure. Scree plot following exploratory factor analysis. eTable 1. Variables included in the exploratory factor analysis. eTable 2. Over-dispersion data for each outcome. eTable 3. Neighborhood-level descriptive characteristics of racial/ethnic density, fragmentation and diversity, by racial/ethnic group. eTable 4. Rotated factor loadings following EFA. [file jamapsychiatry-75-75-s001.pdf]

Supplementary Online Content

Richardson R, Hameed Y, Perez J, Jones PB, Kirkbride JB. Association of environment with the risk of developing psychotic disorders in rural populations: findings from the Social Epidemiology of Psychoses in East Anglia study. *JAMA Psychiatry.* Published online November 29, 2017. doi: 10.1001/jamapsychiatry.2017.3582.

eAppendix. Online-only text.

eFigure. Scree plot following exploratory factor analysis.

eTable 1. Variables included in the exploratory factor analysis.

eTable 2. Over-dispersion data for each outcome.

eTable 3. Neighborhood-level descriptive characteristics of ethnic density, fragmentation and diversity, by ethnic group.

eTable 4. Rotated factor loadings following EFA.

This supplementary material has been provided by the authors to give readers additional information about their work.

© 2017 Richardson R et al. *JAMA Psychiatry.*

eAppendix. Online-Only text

Neighborhood-level exposure constructs of the social and built environment were derived from 29 items entered into an exploratory factor analysis. We obtained 24 items from the UK 2011 census related to neighborhood ethnic and social composition, residential stability, social isolation, housing tenure, household composition, population density, deprivation and inequality. Multiple deprivation was measured as the proportion of households per neighborhood deprived on 2 or more of 4 domains in the UK 2011 Census (employment, education, living environment, health), as previously described[4]. We calculated two measures of inequality. First, we estimated multiple deprivation in smaller geographical units known as “lower super output area” [LSOA] (N=1475), nested within each statistical ward (median: 3; IQR: 1-7). Inequality in deprivation across the LSOA within each neighborhood was estimated using a Gini-like methodology, ranging from 0 (perfect equality) to 100 (perfect inequality). Second, similarly, we estimated socioeconomic inequality in each statistical ward, based on the population distribution across SES categories, as described above. We also included three measures of the built environment (% green space, % blue space, % non-residential buildings) from the Office of National Statistics (ONS) Generalized Land Use Database[26], and two measures of accessibility to services, via data from the ONS Journey Time Statistics Collection[27] (mean journey time to the nearest town, weighted from LSOA-level data to each statistical ward, based on population size) and the UK 2011 Census (mean distance to nearest workplace).

© 2017 Richardson R et al. *JAMA Psychiatry.*

eFigure. Scree plot following exploratory factor analysis

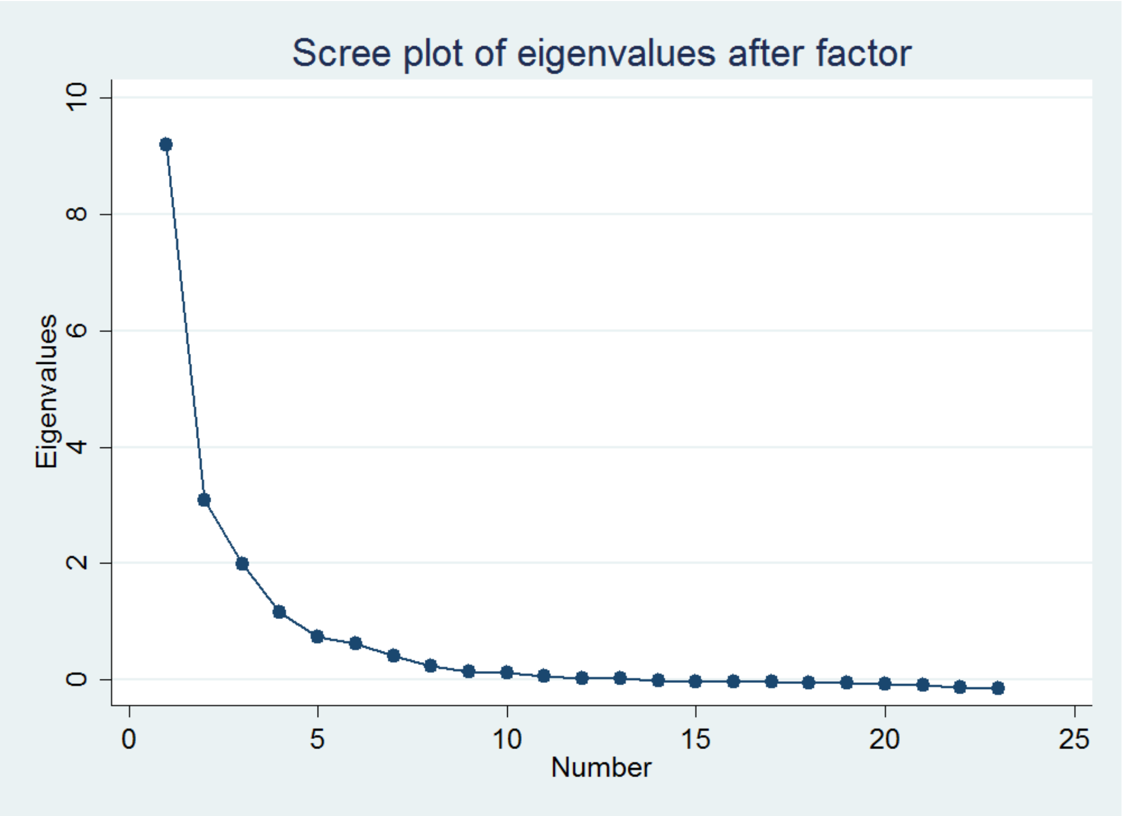

Legend: Following EFA, a scree plot suggested a four-factor solution led to the optimum trade-off between number of factors and variance explained. These four factors explained 90% of the variance in neighborhood-level items.

© 2017 Richardson R et al. *JAMA Psychiatry.*

eTable 1 Variables included in the exploratory factor analysis

No.	Variable label	Description	Source - Table number	Excluded from EFA?
1	Population density	Population density (people per square mile)	2011 Census – PHP01	
2	People per household	Number of people per household	2011 Census – PHP01	Yes: poor KMO in EFA (0.58)
3	% age 16-35 density	% population aged 16-35 years	2011 Census – QS103EW	
4	% single household	% people living alone without dependent children	2011 Census – KS105EW	
5	% single parent household	% single parent households with dependent children	2011 Census – KS105EW	
6	% Non-cohabiting adults	% people 16+ years not cohabiting	2011 Census – KS104EW	
7	% multiple deprivation	% households deprived on 2+ (of 4) Census domains	2011 Census – QS119EW	
8	% Inequality	% deprivation inequality within electoral wards (Gini method)	2011 Census – QS119EW	
9	% SES inequality	% inequality in SES, 16-74 years, excluding students (Gini method)	2011 Census – QS607EW	Yes – poor KMO in EFA (0.57)
10	% Socially rented housing	% people living in social rented housing	2011 Census – QS403EW	
11	% Privately rented housing	% people living in private rented housing	2011 Census – QS403EW	
12	% No car ownership	% households with no car	2011 Census – KS404EW	
13	% No central heating	% households with no central heating	2011 Census – QS415EW	
14	% BME	% population of BME status	2011 Census – KS201EW	Yes: 0.98 correlation with #15 & #16
15	% Ethnic diversity	Ethnic diversity across 18 ethnic groups (Reciprocal Diversity Index)	2011 Census – KS201EW	
16	% non-UK born	% population not born in the UK	2011 Census – QS803EW	Yes: 0.99 correlation with #17
17	% non-UK identity	% population not self-identifying as from UK	2011 Census – KS202EW	
18	% Recent migrants (mb)	% of immigrants who arrived in last 2 years (migrant base)	2011 Census – QS803EW	
19	% Recent migrants (UKb)	% of immigrants who arrived in last 2 years (UK population base)	2011 Census – QS803EW	Yes: 0.95 correlation with #18
20	% Poor English	% people aged 3+ years with English rated as poor	2011 Census – DC2803EW	
21	% In-migration (total)	% population who moved into neighborhood in previous 12 months	2011 Census – MM01CUK	
22	% In-migration (overseas)	% total population who moved into the neighborhood from outside UK in previous 12 months	2011 Census – MM01CUK	
23	% Out-migration	% population who moved out of neighborhood in previous 12 months	2011 Census – MM01CUK	
24	Rural-Urban category	ONS Rural-urban classification (urban/town&fringe/rural)	2014 Rural-Urban Classification	Yes: no substantial factor loading
25	Mean distance to work	Mean distance (km) travelled to work by working population	2011 Census – QS702EW	
26	Journey time to town	Average journey time in minutes to nearest town center by car	ONS Journey Time Statistics[27]	
27	Area non-domestic buildings	Area of non-domestic buildings (km^2)	ONS Generalized Land Use Database[26]	
28	Area greenspace	Area of greenspace (km^2)	ONS Generalized Land Use	

© 2017 Richardson R et al. *JAMA Psychiatry.*

			Database[26]	
29	Area water	Area of water (km^2)	ONS Generalized Land Use Database[26]	Yes: no substantial factor loading

Abbreviations: EFA, Exploratory Factor Analysis; BME, Black Ethnic Minority. KMO: Kaiser-Meyer-Olkin sampling adequacy. Census data accessed from http://www.nomisweb.co.uk/census/2011

© 2017 Richardson R et al. *JAMA Psychiatry.*

eTable 2: Over-dispersion data for each outcome

Outcome	N	Mean	Variance
All psychoses (F20-33)	61,803	0.01	0.01
Non-affective psychoses (F20-29)	61,803	0.01	0.01
Affective psychoses (F30-33)	61,803	<0.01	<0.01

Legend: For all outcomes, there was no evidence that variance in the outcome, exceeded the mean, suggesting data did not violate this Poisson assumption

© 2017 Richardson R et al. *JAMA Psychiatry.*

eTable 3: Neighborhood-level descriptive characteristics of ethnic density, fragmentation and diversity, by ethnic group

Neighborhood-level exposure	Median	IQR	Correlation with:
**Ethnic density (%)**			*Ethnic fragmentation*
White British	94.5	88.9 – 96.5	-0.12*
White other	2.9	2.0 – 5.3	-0.18*
Mixed White and Black Caribbean	0.3	0.2 – 0.5	-0.60*
Mixed other	0.8	0.5 – 1.2	-0.56*
Indian	0.4	0.2 – 0.9	-0.67*
Pakistani	0.2	0.1 – 0.4	-0.42*
Bangladeshi	0.2	0.1 – 0.3	-0.67*
Black African	0.3	0.1 – 0.6	-0.72*
Black Caribbean	0.2	0.1 – 0.3	-0.56*
Arabic	0.1	0.1 – 0.2	-0.68*
Any other ethnic group	0.8	0.4 – 1.4	-0.47*
Kruskal-Wallis χ^2 10d.f.; p-value	2955.6; p<0.001		
**Ethnic fragmentation (%)**			*Ethnic diversity*
White British	19.8	15.9 – 24.4	0.12*
White other	22.6	18.1 – 27.2	-0.23*
Mixed White and Black Caribbean	53.4	44.3 – 63.8	-0.35*
Mixed other	34.6	28.0 – 42.6	-0.43*
Indian	59.9	46.4 – 72.7	-0.51*
Pakistani	78.4	64.7 – 87.2	-0.60*
Bangladeshi	82.8	70.9 – 89.6	-0.47*
Black African	65.4	51.0 – 77.6	-0.55*
Black Caribbean	65.1	53.0 – 71.2	-0.42*
Arabic	79.7	66.6 – 88.7	-0.64*
Any other ethnic group	39.9	31.8 – 49.5	-0.43*
Kruskal-Wallis χ^2 10d.f.; p-value	3027.2; p<0.001		
**Ethnic diversity (z-score)**			*Ethnic density*
White British	-0.3	-0.6 – 0.2	-0.87*
White other	-0.3	-0.6 – 0.2	0.87*
Mixed White and Black Caribbean	-0.3	-0.6 – 0.4	0.21*
Mixed other	-0.3	-0.6 – 0.3	0.82*
Indian	-0.2	-0.5 – 0.5	0.51*
Pakistani	0.1	-0.4 – 0.9	0.36*
Bangladeshi	0.0	-0.5 – 0.8	0.26*
Black African	-0.2	-0.5 – 0.5	0.56*
Black Caribbean	-0.1	-0.4 – 0.7	0.25*
Arabic	0.0	-0.4 – 0.9	0.52*
Any other ethnic group	-0.3	-0.6 – 0.3	0.84*
Kruskal-Wallis χ^2 10d.f.; p-value	109.4; p<0.001		

Footnote: Median values of ethnic density, fragmentation & diversity by ethnic group, across 530 neighborhoods in the SEPEA catchment area. Ethnic fragmentation describes own-group ethnic fragmentation and is the % of an ethnic group which would have to move to another part of the neighbourhood to achieve perfect ethnic integration: higher values indicate lower own-group fragmentation/higher segregation from the remainder of the population. Data confirms higher own-group ethnic density and lower fragmentation in the white British group than ethnic minority groups. White British, white other and people of mixed ethnic groups tended to live in less ethnically diverse neighborhoods than other ethnic groups. For all ethnic groups, higher own-group ethnic density was negatively correlated with ethnic fragmentation (i.e. positively correlated with ethnic segregation), though the magnitude of this effect varied. Own-group ethnic fragmentation was also correlated with neighborhood-level ethnic diversity. Here, for the white British group, greater fragmentation was associated with greater ethnic diversity, for all other groups greater fragmentation (i.e. lower segregation) was associated with greater neighborhood ethnic diversity. Finally, greater own-group ethnic density was strongly negatively correlated with neighborhood-level ethnic diversity in the white British group, but strongly positively correlated with ethnic diversity for all other ethnic groups.
d.f.: degrees of freedom; *p<0.05

© 2017 Richardson R et al. *JAMA Psychiatry.*

eTable 4: Rotated factor loadings following EFA[a]

Variable	Factor			
	1 Ethnic diversity	2 Deprivation	3 Urbanicity	4 Social isolation
% non-UK identity	0.92			
% In-migration (overseas)	0.91			
% In-migration (total)	0.83			0.43
% Ethnic diversity	0.81			
% Age 16-35 density	0.78			
% Out-migration	0.74			0.48
% Recent migrants (mb)	0.74			
% Privately rented housing	0.71			0.48
% Multiple deprivation		0.82		
% Poor English	0.53	0.64		
% Single parent household		0.63		
% Socially rented housing		0.61		
% No car ownership		0.58		0.60
% Non-Cohabiting adults	0.41	0.51		0.64
% Inequality		-0.41		
Area greenspace			-0.82	
Area non-domestic buildings			-0.68	
Mean distance to work			-0.67	
Journey time to town			-0.61	
Population density			0.50	0.41
% No central heating				0.49
% Single household				0.77

[a]For clarity, only loadings ≥±0.4 are displayed. Varimax rotation used.

© 2017 Richardson R et al. *JAMA Psychiatry.*
